# A Novel Data Reduction Approach for Structural Health Monitoring Systems

**DOI:** 10.3390/s19224823

**Published:** 2019-11-06

**Authors:** Hamed Bolandi, Nizar Lajnef, Pengcheng Jiao, Kaveh Barri, Hassene Hasni, Amir H. Alavi

**Affiliations:** 1 Department of Civil and Environmental Engineering, Michigan State University, East Lansing, MI 48824, USA; bolandih@msu.edu (H.B.); lajnefni@egr.msu.edu (N.L.); hasniha1@msu.edu (H.H.); 2 Ocean College, Zhejiang University, Zhoushan 316021, China; 3 Department of Civil and Environmental Engineering, University of Pittsburgh, Pittsburgh, PA 19104, USA; KAB421@pitt.edu

**Keywords:** data reduction, strain data, probability theory, steel plate, structural health monitoring

## Abstract

The massive amount of data generated by structural health monitoring (SHM) systems usually affects the system’s capacity for data transmission and analysis. This paper proposes a novel concept based on the probability theory for data reduction in SHM systems. The beauty salient feature of the proposed method is that it alleviates the burden of collecting and analysis of the entire strain data via a relative damage approach. In this methodology, the rate of variation of strain distributions is related to the rate of damage. In order to verify the accuracy of the approach, experimental and numerical studies were conducted on a thin steel plate subjected to cyclic in-plane tension loading. Circular holes with various sizes were made on the plate to define damage states. Rather than measuring the entire strain response, the cumulative durations of strain events at different predefined strain levels were obtained for each damage scenario. Then, the distribution of the calculated cumulative times was used to detect the damage progression. The results show that the presented technique can efficiently detect the damage progression. The damage detection accuracy can be improved by increasing the predefined strain levels. The proposed concept can lead to over 2500% reduction in data storage requirement, which can be particularly important for data generation and data handling in on-line SHM systems.

## 1. Introduction

Structural health monitoring (SHM) is an emerging field which has received notable attention in recent years. In general, the goal of SHM is to monitor the integrity of structures [1,2,3,4]. Strain gauges have been widely used in the fields of civil, mechanical and aerospace engineering to monitor the health status of structural systems. Wu et al. [5] developed damage identification method for concrete continuous girder bridges based on spatially-distributed long-gauge strain sensing. Zymelka et al. [6] designed a low-cost graphite-based strain sensors that could be arranged in the form of an array. Yin et al. [7] presented and fabricated highly durable ternary conductive nanocomposite with elastic strain performance as a stretchable strain gauges. Nie et al. [8] developed a simple-structured and low-cost grapheneon flexible strain gauge which can detect different strain levels of structural variation One of the challenges with deploying SHM systems that rely on strain gauges is that the generated sensory data can easily overwhelm the capacity of the system for data transmission and analysis [9,10]. This issue can be exacerbated in situations such as extreme events where the communication bandwidth may become very limited. Arguably, reducing communication rate in sensor networks when designing and implementing a real-time SHM system can enhance its general performance. Moreover, this will alleviate the power consumption problems in the SHM systems based on wireless sensor networks (WSNs) [11]. A number of studies have been conducted to explore a balance between lowering communication rate and maintaining the quality of the collected data. Park et al. [12] developed a novel method for reducing data in SHM systems via choosing the necessary damage detection features. Jiang et al. [13] proposed a framework for clustering-based data collection. To improve prediction accuracy, Carvalho et al. [14,15] presented a multivariate spatial approach to enhance the prediction accuracy in WSNs data reduction. The linear predictor-based lossless data is another compression method developed for compressing vibration sensor data. This system can be implemented to achieve better compression performance with the use of an auto-regressive (AR) model-based linear predictor [16]. Tan and Wu [17] proposed a hierarchical lean mean square error (HLMS) prediction algorithm and protocol to increase the convergence rate during the preliminary phase of the algorithm and to decrease the amount of real-time data transmitted to the sink. Li et al. [11] designed and implemented an on-line SHM system focused on streamlined data flow. Wu et al. [18] presented a data compression framework for reducing the communication exchanges. A lean mean square error (LMS) adaptive strategy was developed by Santini and Romerin [19] to reduce data in WSNs. Also, an in-network data reduction method was presented by Pattem and Silberstein [20] based on the integrated data compression and routing methods. Kolo et al. [21] presented a lightweight adaptive data compression method for WSNs. The enhanced compression achieved by the algorithm decreases the network burden which results to fewer collisions and retransmissions. He et al. [22] and He and Li [23] developed spatial and temporal data compression algorithms for the reduction of Big Data in WSNs. Lada et al. [24] proposed a new criterion, labeled as relative reconstruction error (RRE) for data reduction in WSNs. Jeong et al. [25] further studied the efficacy of RRE for data reduction. Bukkapatnam et al. [26] presented a new data reduction method which was more efficient than the common de-noising or RRE approaches. Yoon and Shahabi [27] presented a clustered aggregation (CAG) algorithm to reduce the number of wireless transmissions. Peckens and Lynch [28] proposed a cochlea-inspired sensor capable of large data compression. Heo et al. [29] developed a software to compress the acceleration signals. Chae et al. [30] used data filtering rule where a group of data in sensor networks are removed and replaced by the mean value. Chu et al. [31] proposed an approximate approach called Ken to minimize communication from sensing nodes to the server. Wang et al. [32] proposed BigReduce which is a cloud-based infrastructure health monitoring method within the IoT framework. They reduced the burden of big data processing at the base station. A fidelity data collection framework was developed by Wu et al. [33] to decrease the number of the active sensor nodes. Machine learning methods are proved to be helpful for data mining and data reduction in sensor networks [34,35,36,37,38]. In this arena, Avci et al. [39] presented a convolutional neural networks (CNN) technique to minimize the wireless computational time and power consumption. Recently, Chakrabartty et al. [40], Lajnef et al. [41,42] and Alavi et al. [43] studied piezo-floating-gate (PFG) self-powered wireless sensors that integrate piezoelectric sensors with floating-gate computational circuits [44]. The floating-gates act as a series of memory cells that can compress and store the load history profiles in terms of cumulative time at specific preset voltage levels [40,41]. Alavi et al. [44,45] and Hasni et al. [46] proposed machine learning methods to detect structural damage using the compressed data collected by the PFG sensors. However, the studies focused on the PFG sensing are specifically designed for systems that combine energy harvesting and floating-gates technologies. 

The overarching goal in this study is to develop a generic data reduction method for SHM systems that rely on strain data collected by conventional strain gauges. The proposed method based on the probability theory uses the cumulative duration of strain events at different predefined strain levels for damage detection. This way, the need to measure and store the entire strain response is eliminated, which will arguably result in reducing the communication rates, data storage and analysis. A series of experimental and numerical studies was conducted on a thin steel plate subjected to cyclic in-plane tension to validate the proposed method. 

## 2. The Proposed Data Reduction Method

**Figure 1:**
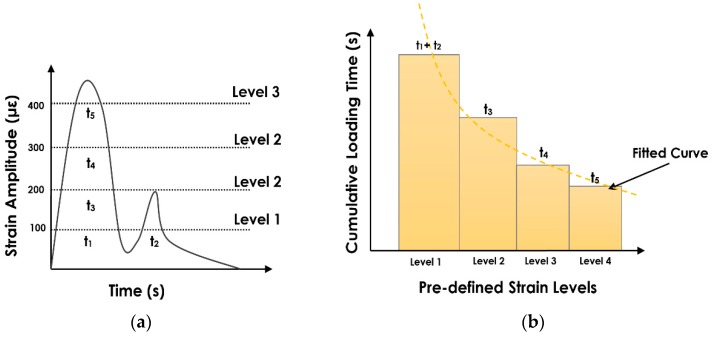
The cumulative loading time estimation. (**a**) strain amplitude and (**b**) measured cumulative loading time.

The cost-effective, sensitive and easy-to-install strain gauges have been extensively used in the SHM arena for damage detection. One of the significant challenges in monitoring the health of large structures is to store and interpret the large amount of data generated by the strain gauges. The method proposed in this study deals with reducing the amount of strain data that needs to be transmitted and analyzed. Inspired by the level crossing cumulative time counting approach [32,33,34,35], the proposed methodology is based on a “relative damage” approach. It is called a relative damage detection approach because it dies not demand for directly measuring the entire strain response. In fact, the method explores the relationship between the indicative features from the strain patterns and the damage progression. In this method, only the duration of strain events at a preselected level discretization is cumulatively measured. This procedure is schematically presented in Figure 1. The first step is to predefine the strain thresholds which is based on an approximation of the minimum and maximum of the experienced strains in the investigated structure. Assuming that the structure will experience strains between 0 to 450 microstrain (µε), we can select four strain levels (level 1 = 100 µε, level 2 = 200 µε, level 3 = 300 µε, and level 4 = 400 µε) within this range. As seen in Figure 1b, the cumulative time at specific predefined strain thresholds is then calculated for a random excitation given in Figure 1a. Accordingly, the cumulative loading times for the Levels 1 to 4 are equal to t1 + t2, t3, t4, and t5, respectively. The obtained cumulative loading time in Figure 1b incorporates compressed data representing the actual strain response. In other words, the actual strain response can be reconstructed merely with the cumulative duration of strain events. Obviously, considering more predefined strain levels will lead to constructing a cumulative time histogram that contains more information about the original strain.

In order to characterize the obtained histograms, a distribution function can be fitted to the cumulative time distributions as shown in Figure 1b. The hypothesis is that damage progression can be inferred using these cumulative probability distributions. In other words, the cumulative probability distributions corresponding to the strain data for each damage state will have a unique shape. Accordingly, a number of cumulative probability distributions can be considered as follows:

Rayleigh distribution:

(1) $$F_{\mathrm{Rayleigh}}\left(\varepsilon \right)=1-e^{\frac{-x^{2}}{2\sigma^{2}}},$$

where *σ* is the standard deviation of the distribution.

Shifted Gompertz distribution:

(2) $$F_{\mathrm{Gompertz}}\left(\varepsilon \right)=\left(1-e^{-bx}\right)e^{-\eta e^{-bx}},$$

where *b* > 0 is the scale parameter and η > 0 is the shape parameter of the shifted Gompertz distribution.

Weibull distribution:

(3) $$F_{\mathrm{Weibull}\;}\left(\varepsilon \right)=\{\begin{array}{cc} 1-e^{-{\left(\frac{x}{\lambda }\right)}^{k}} & x\geq 0\\ 0 & x\geq 0 \end{array},$$

where k and *λ* are, respectively, the shape and scale parameters of the distribution.

Fréchet distribution:

(4) $$F_{\mathrm{Fréchet}\;}\left(\varepsilon \right)=e^{-\left(\frac{x-m}{s}\right)-\alpha },$$

where *α* > 0 is a shape parameter, *m* (the minimum) is a location parameter and *s* > 0 is a scale parameter.

Gaussian cumulative density function:

(5) $$F_{\mathrm{Gaussian}\;}\left(\varepsilon \right)=\frac{\alpha }{2}\left[1-erf\left(\frac{\varepsilon -\mu }{\sigma \sqrt{2}}\right)\right],$$

where, *μ*, *σ*, and *α* are the mean of the cumulative time distribution, standard deviation of the cumulative time distribution and total cumulative time of the event, respectively. *erf* denotes the Gauss error function. 

To verify the hypothesis, experimental and numerical studies were carried out in this study. For the experimental study, several strain gauges were installed on a thin steel plate. The strain values for various damage states were recorded. While the strain gauges are sensing the strain, the proposed method measures the cumulative time of the applied strain. The same procedure was followed for the numerical simulations. Then, MATLAB was used to automatically perform the following tasks:

- Measuring the cumulative times at designed levels from the strain signals
- Fitting a distribution function to the cumulative time histograms
- Calculating the cumulative distribution features for damage detection

## 3. Experimental Study

**Figure 2:**
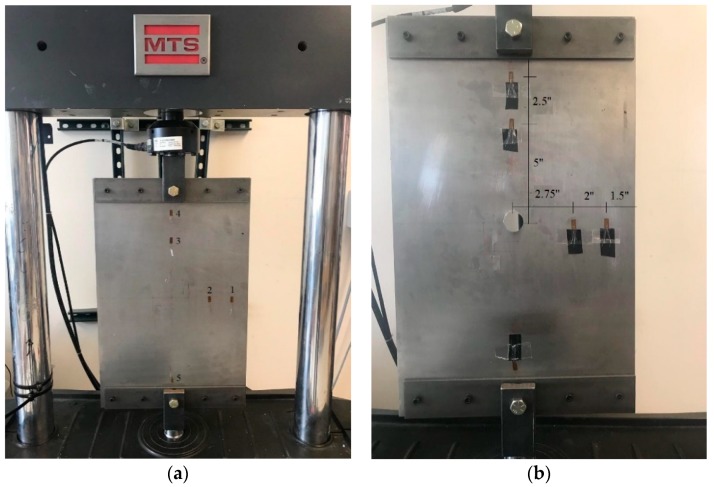
Test setup and strain gauge locations. (**a**) Intact plate and (**b**) Damaged plate.

An experimental study was conducted to determine the strain in each damage state. A uniaxial tension mode was considered for testing the steel plate (A-32 steel grade). The length, span, width, and thickness of the steel plate are, respectively, 508 mm (20 inches), 406.4 mm (16 inches), 304.8 mm (12 inches), and 0.8 mm (1/32 inch). Figure 2 shows the specimen and strain gauge locations. A similar experimental setup to the previous studies [35] was considered in this work. Accordingly, four thick steel plates, 50.8 mm × 304.8 mm (2 inches × 12 inches), were installed at the top and bottom of the plate. Eight screws and two bolts were used at the center of plate to restrain the horizontal displacement, rotation and to connect the specimen to the MTS machine. The strain gauge locations were determined based on a preliminary 3D finite element (FE) analysis to find the regions with maximum stress and strain concentration. ABAQUS/CAE 2017 was used for this purpose. Circular holes with different diameter sizes (D) were created manually at the middle of the plate to introduce the damage sates (DS):

- Intact: D = 0 mm
- Damage state 1 (DS1): D = 12.7 mm (0.5 inch)
- Damage state 2 (DS2): D = 25.4 mm (1 inch)

Two strain gauges were installed on the plate along the x-axis near the potential damage zone. Another strain gauge was mounted along the y-axis almost far from the damage zone. Moreover, two strain gauges were installed at the top and bottom part of the plate as reference nodes for the boundary conditions. The chosen strain gauges were one-axis linear gauges with resistance of 350 Ω, gauge factor of 2.04, and temperature compensation of *α* = 10.8 [10–6 K]. CC-33A epoxy was used to attach the strain gauges to the plate. A half-sine cyclic displacement (at 2 Hz and 0.08 mm amplitude) was applied to the specimen edge.

**Figure 3:**
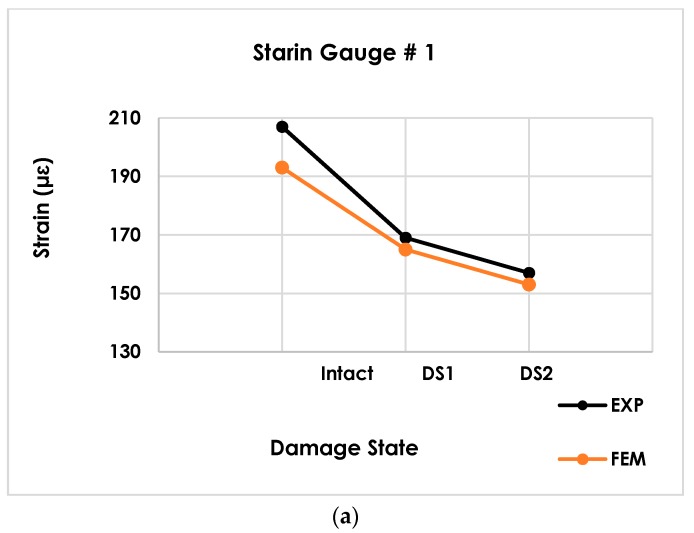

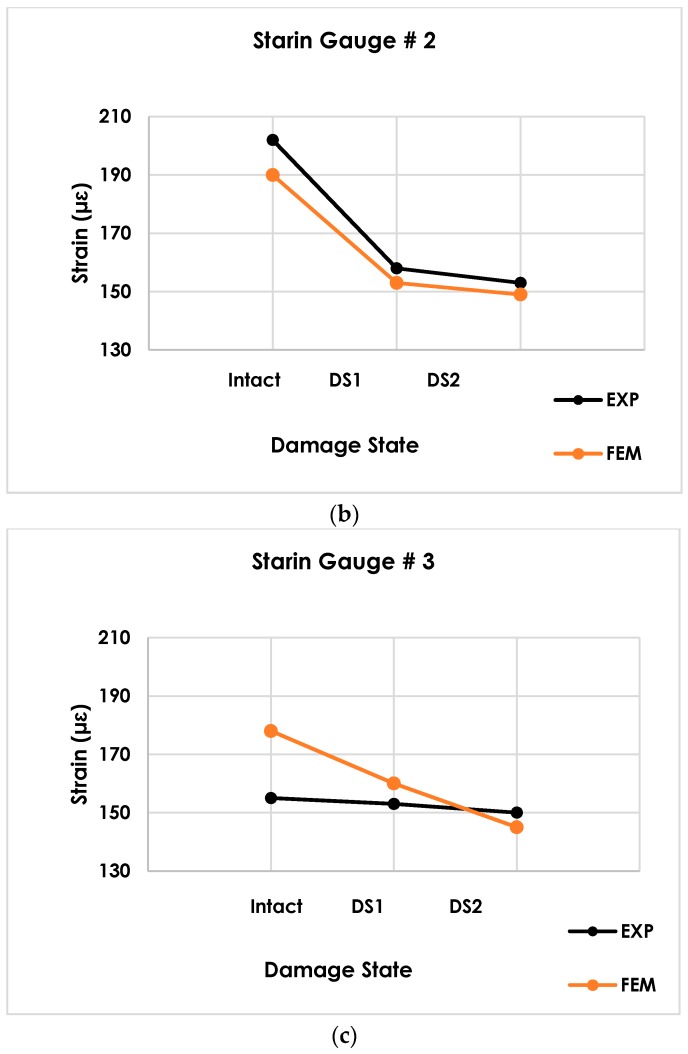
A comparison of the maximum strain measured by the strain gauges. (**a**) strain gauge # 1 (**b**) strain gauge # 2, and (**c**) strain gauge # 3.

Figure 3 shows the maximum strain values measured by the strain gauges for different damage states in both experimental and FE models. At locations 1 to 3, the strain decreases as the damage progresses. (Figure 3a,b). Comparing strain gauges 1 and 2 with strain gauge 3, it can be observed that the strain reduction rate in the x-direction is higher than the y-direction. This is expected because strain gauges 1 and 2 are located closer to the damage zone. The maximum strain values measured by strain gauges 1–3 were, respectively, 207, 202 and 155 µε in the intact mode. The minimum strain values measured by strain gauges 1–3 were, respectively, 157, 153 and 150 µε in the DS2 mode. An acceptable agreement is seen between the experimental and FE results.

## 4. Numerical Study

**Figure 4:**
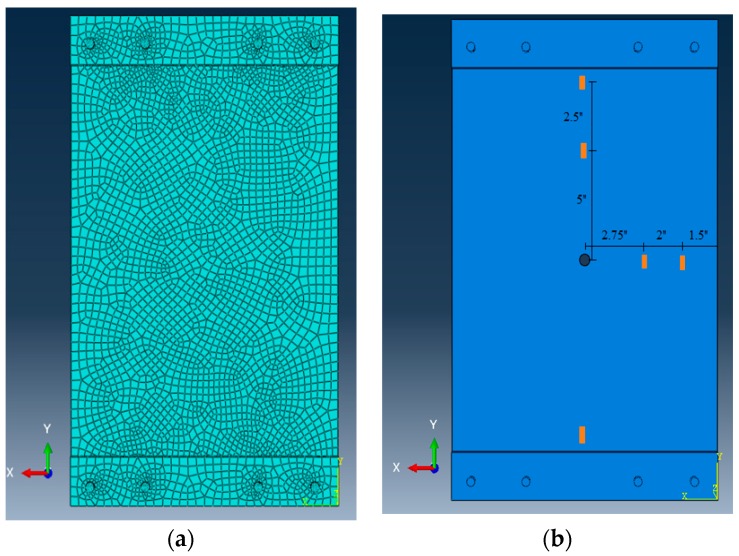
The FE analysis of the plate: (**a**) meshing and (**b**) sensing node locations.

In order to numerically study the response of the plate, three FE models were established. The FE models provided information about the regions with maximum stress and strain concentration, as well as the size of the damaged-induced stress zone. These results were then used to determine the optimal location of the sensors on the plate. A dynamic implicit analysis was conducted. Longitudinal partitions with the same size as the strain gauge were created to represent the sensing nodes. The FE mesh and sensing node locations are presented in Figure 4. Damage states were defined similar to the previous step. Also, a mesh refining was used. The details of the steel plate FE model are as follows:

- Total of 3129 C3D8R elements
- Elastic modulus (E) = 200 GPa
- Poisson’s ratio (ν) = 0.3
- Density = 7800 kg/m^3^.

**Figure 5:**
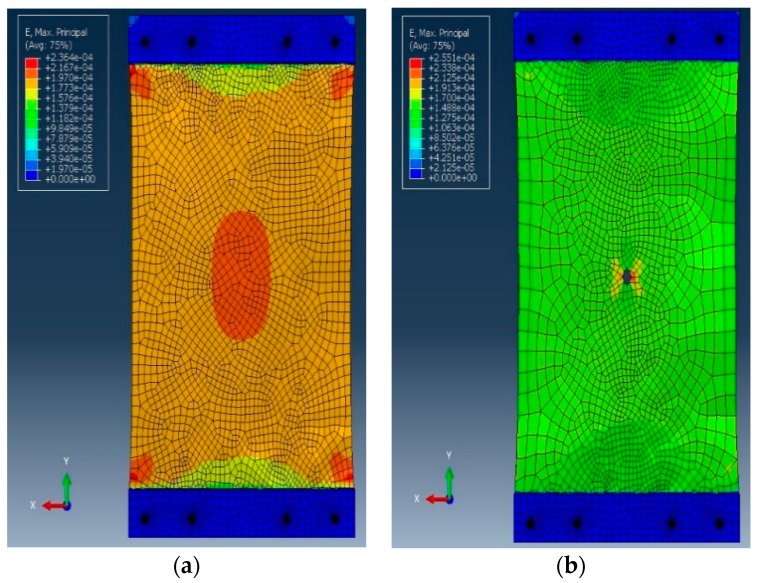

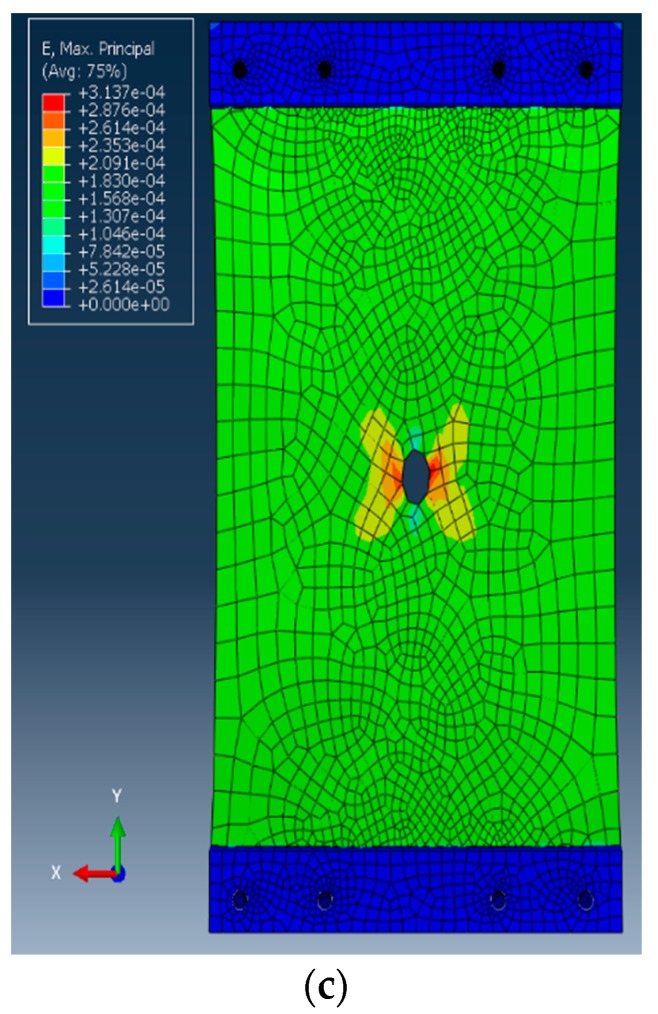
The FE results for different damage states. (**a**) Intact plate (**b**) damage sates (DS)1 (D = 12.7 mm), and (**c**) DS2 (D = 25.4 mm).

Figure 5 shows the FE results for different damage states. The maximum strain around circular hole increased from 255 µε to 313 µε between the DS1 (0.5 inch) and DS2 (1 inch). The strains sensed at sensing nodes 1, 2 and 3 decreases slightly with damage progression, which is in agreement with the experimental observations. The maximum strain values at sensing nodes 1, 2 and 3 were, respectively, 193, 190 and 178 µε, for the intact mode. The minimum strain values measured at sensing nodes 1, 2 and 3 were, respectively, 153, 149 and 145 µε in the DS2 mode.

## 5. Damage Growth Detection Using the Proposed Data Reduction Method 

**Figure 6:**
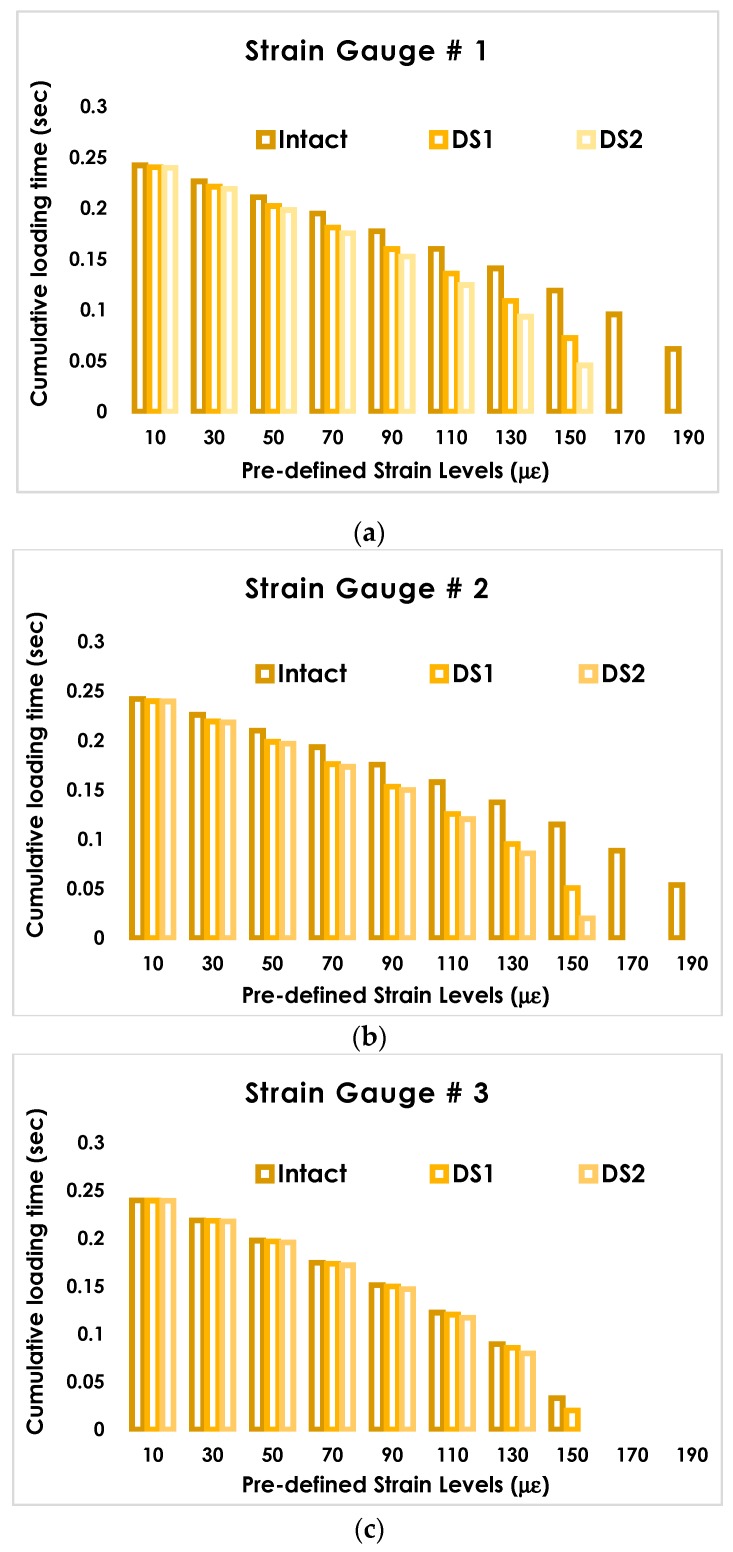
Cumulative time versus predefined strain levels for the intact and damaged states. (**a**) strain gauge # 1 (**b**) strain gauge # 2, and (**c**) strain gauge # 3.

The proposed data reduction method is based on cumulatively measuring the duration of strain events at preselected levels. Table 1 presents the preselected strain levels in this study. The strain levels were increased by steps of 20 µε for 10 levels starting from 10 µε, which was equal to the minimum strain in the plate. Following the procedure given in Section 2, the durations of events at predefined strain levels were calculated for each sensing node and damage state. Since the strains obtained from the FE simulations were almost identical to those from the experimental study, the cumulative times were only presented for the experimental data. Figure 6 shows the cumulative time histograms for different damage states at the predefined strain levels for strain gauges 1–3. 

The next step was to fit a cumulative distribution function to the distributions. This task was performed using nonlinear curve fitting via “lsqcurvefit” function in Matlab. The implementation of this function in Matlab for each of the obtained histograms is fairly fast (<2 s). After trying the cumulative probability distributions presented in Section 2, it was found that the Gaussian cumulative density function (CDF) provides a better and more consistent fit to the to the cumulative duration of strain events. Thus, this function was considered for the characterization of the strain data. Subsequently, the corresponding *μ* and *σ* values for each damage scenario were obtained. Then, *μ* and *σ* were used for plotting the following probability density function (PDF):

(6) $$PDF\left(\varepsilon \right)=\frac{1}{\sigma \sqrt{2\pi }}e^{-\frac{\left(\varepsilon -\mu \right)}{2\;\sigma^{2}}}.$$

The idea here is that there is a direct relationship between the changes of the PDFs and damage states. This can be considered as a major advantage in detecting damage because the focus can be placed on the strain relative variations. In other words, it is not necessary to directly measure the damage. Instead, relative changes of the PDFs over time will provide an insight into the damage progression. The PDFs carry the critical information about the damage effect which is already measured by the strain gauges. If a sensing node is closer to the damage zone, there would a more significant changes in the corresponding PDF patterns. 

**Figure 7:**
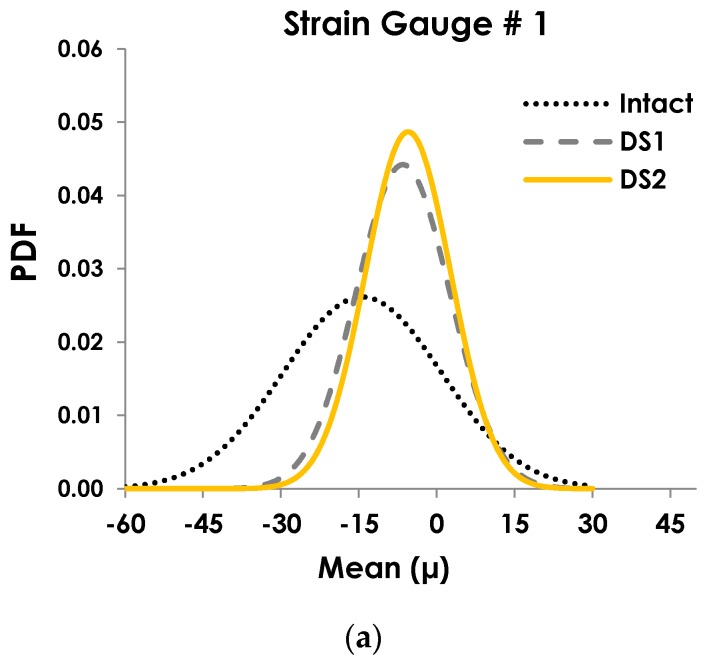

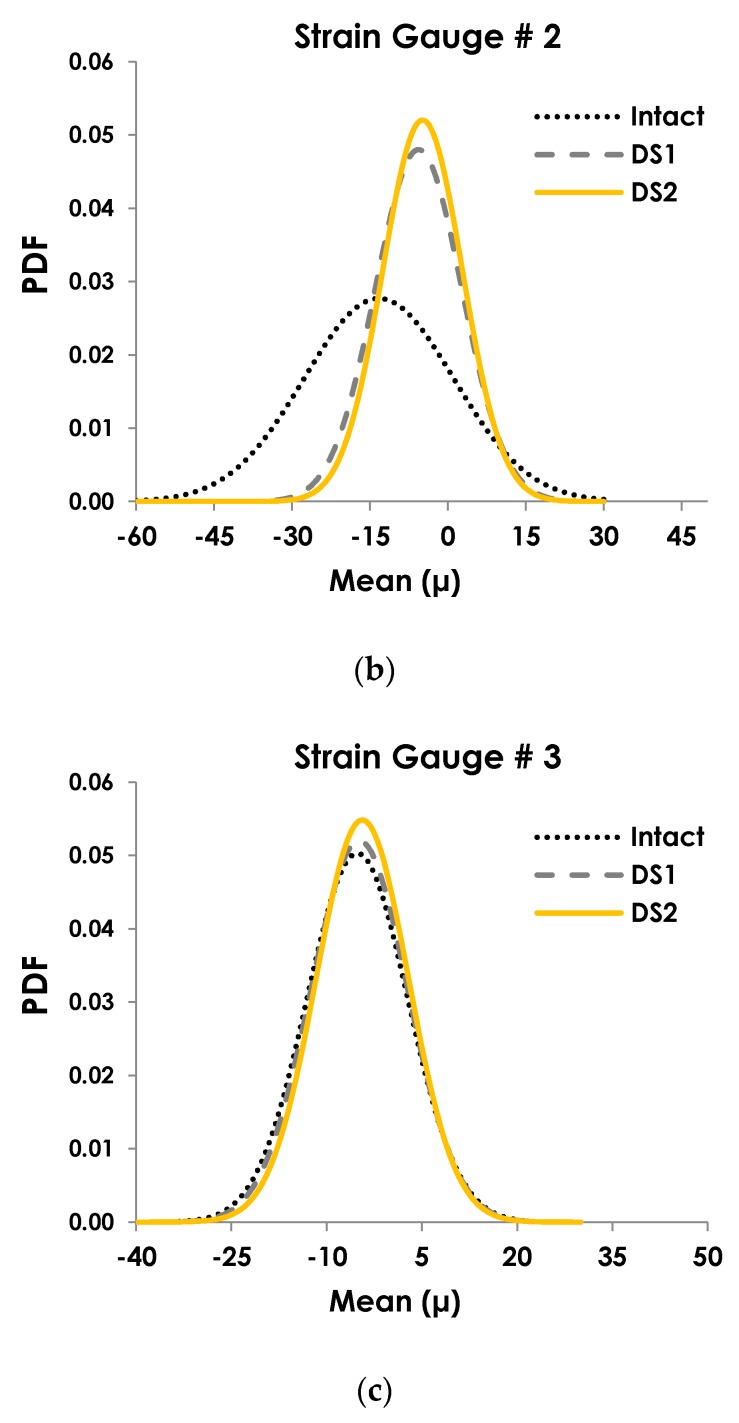
The relationship between the probability density function (PDF) patterns and damage progression. (**a**) strain gauge # 1 (**b**) strain gauge # 2, and (**c**) strain gauge # 3.

The PDF plots corresponding to each strain gauge are shown in Figure 7. The parameter µ in the horizontal axis denotes the mean of the cumulative time distribution and not the pre-defined strain levels. This parameter was calculated by fitting the CDF curve to the cumulative time histograms. While the pre-defined strain levels are positive values, the nonlinear curve fitting can return the positive or negative µ values for a given cumulative time bin. *µ* values close to zero indicate that the corresponding cumulative time bins have a distribution similar to a normal distribution. However, it is seen that there is a sound correlation between the PDFs shape and damage progression. Referring to Figure 7a,b, PDFs corresponding to strain gauges 1 to 2 can detect the damage progression efficiently. Increasing the size of the damage leads to increasing in *μ* and decreasing *σ*. This will shift the PDFs to right and contracts it. The same observation is true for strain gauge 3 (Figure 7c) with less obvious changes in the PDF shape. For all sensors, the PDF values shown in y-axis are increasing. Note that strain gauges 1 and 2 are located near the damage zone and expectedly experience higher variations of the strains when damage progresses. As seen in Figure 3a,b, the strain amplitudes are lower for DS1 and DS2 than those for the intact mode, which results in lower recorded cumulative time. If a CDF is fitted to a histogram with smaller cumulative time bins, the algorithm will return higher *µ* and lower *σ*, and therefore, a higher PDF value. This observation will be inverse if the strain values at a specific point increase due to the damage propagation. 

## 6. Discussion

The proposed method cannot only be used for damage detection but can also be used for damage localization. To this aim, the variation of the *µ* and *σ* values with damage progression can be analyzed. Table 2 shows the variation of *µ* and *σ* for the Intact-DS1 and DS1–DS2 modes in the experimental study. Referring to this table, the *µ* and *σ* variations are notable for strain gauges 1 and 2 that are located around the hole. The maximum variation (57%) belongs to *µ* of strain gauge 2 when damage progresses from the Intact to DS1 mode. The variations of *σ* seem to have a lower rate for each transitioning mode compared to *µ* for all of the sensors. The other observation is that the variation of *µ* and *σ* for strain gauges 1–3 is remarkably higher for the Intact-DS1 mode than that for the DS1–DS2 mode. Obviously, the detection resolution is dependent on the sensor network design. For larger structures, damage localization will require installing more sensors to achieve a better resolution. In such cases, it is feasible to fuse the information contained in the PDFs corresponding to various sensing nodes to improve the damage detection and localization accuracy. 

Besides, the beauty salient feature of this method is that there is no need to record and analyze the entire strain response for SHM. Therefore, it can be used for a more efficient data generation and data handling in an on-line SHM system. For instance, in the current study, storing a full cycle of strain wave at 2 Hz loading frequency required eight kilobytes (KB) of memory. On the other hand, the memory requirement for the cumulative time event data for the strain thresholds defined in Table 1 was reduced to approximately 300 bytes. This is equivalent to a 2667% reduction in storage requirement. One may consider 20 strain levels to achieve a significantly better damage detection accuracy and still save over 1300% in storage capacity. This is especially crucial for wireless communication systems that usually have a limited communication bandwidth. Such aggregated-data collection for in-network data compression can result in reducing the required communication between nodes. It is well-known that the communication unit in WSNs is a major source of power consumption. By reducing the amount of the exchanged information, the proposed data reduction approach can notable save the power consumption. Moreover, due to its low power consumption nature, this approach would be suitable for the WSN nodes operated by batteries. It is worth mentioning that the same concept can be deployed for other monitoring applications such acceleration measurements. 

## 7. Conclusions

A new data reduction procedure has been proposed for the SHM systems. The proposed method is based on the interpretation of the cumulative duration of strain events at different predefined strain levels. The outputs of this procedure are histograms that represent the cumulative time at each of the defined strain thresholds. This method is unique because it does not need to directly measure, store and then analyze the entire strain signal for detecting damage. The proposed relative damage method relates indicative parameters from the strain distributions to the damage rate. Besides, for the considered strain levels in the current study, an over 2500% reduction in storage requirement was achieved. Apparently, more information about the original strain response can be extracted by considering more predefined strain levels. These strain thresholds can be designed based on the desired damage detection accuracy. The more levels there are, the higher the detection performance will be. The algorithm can be easily implemented in any data acquisition (DAQ) system and data loggers. However, the experimental and numerical studies were conducted on a steel plate to evaluate the performance of the data reduction method. A number of cumulative distribution functions were used to characterize the cumulative time distributions for each damage state. It was found that a Gaussian CDF provides a more consistent fit to the to the cumulative duration of strain events. Thus, this function was considered for the characterization of the strain data. Two main parameters of the Gaussian CDF (i.e., *μ* and *σ*) were used to plot the PDF curves. It was observed that the PDF plots shift to right and contract due to damage progression. This means that *μ* increases and *σ* decreases by transitioning from intact to damaged mode. It was shown that the proposed data reduction method can also be used for damage localization. In this, variations of *μ* seem to be a better sign of damage location. However, the results are based on studying a small plate structure. Verification of the long-term performance of the proposed approach for structure, environmental conditions and season variability can be an interesting topic for a future study. Multi-damage cases can also be investigated for a more in-depth evaluation of the proposed method. Furthermore, an optimal sensor placement (OSP) approach can be used to find the most effective sensor configuration.

## Figures and Tables

**Table 1:** The preselected strain levels.

Number	Strain Level (µε)
1	10
2	30
3	50
4	70
5	90
6	110
7	130
8	150
9	170
10	190

**Table 2:** Damage localization using the variation of *µ* and *σ*.

	Variation of *µ*		Variation of *σ*	
	Intact to DS1	DS1 to DS2	Intact to DS1	DS1 to DS2
Strain Gauge 1	−55%	−16%	−41%	−9%
Strain Gauge 2	−57%	−14%	−42%	−8%
Strain Gauge 3	−6%	−10%	−3%	−5%
